# Supplementary material for: Genetic architecture of natural variation in cuticular hydrocarbon composition in Drosophila melanogaster
Source: eLife. 2015 Nov 14;4:e09861. doi: 10.7554/eLife.09861 (PMC4749392; doi:10.7554/eLife.09861)
Supplement: Supplementary file 3. — df: degrees of freedom; P: P-value. DOI: http://dx.doi.org/10.7554/eLife.09861.042 [file elife-09861-supp3.docx]

| Variable | Source | df | Sum of Squares | Mean Square | F Ratio | *P* |
| --- | --- | --- | --- | --- | --- | --- |
| 7,11-C27:2 & 2-Me-C26 | Line | 16 | 0.1137 | 0.0071 | 16.90 | <0.0001 |
|  | Error | 67 | 0.0281 | 0.0004 |  |  |
|  | Total | 83 | 0.1419 |  |  |  |
| 5,9-C27:2 & 9-C27:1 | Line | 16 | 0.7470 | 0.0466 | 35.17 | <0.0001 |
|  | Error | 67 | 0.0889 | 0.0013 |  |  |
|  | Total | 83 | 0.8360 |  |  |  |
|  |  |  |  |  |  |  |
| Excluding DGRP_105 and DGRP_551 | | | | | | |
| Variable | **Source** | **df** | **Sum of Squares** | **Mean Square** | **F Ratio** | ***P*** |
| 7,11-C27:2 & 2-Me-C26 | Line | 14 | 0.0854 | 0.0061 | 14.06 | <0.0001 |
|  | Error | 60 | 0.0260 | 0.0004 |  |  |
|  | Total | 74 | 0.1114 |  |  |  |
| 5,9-C27:2 & 9-C27:1 | Line | 14 | 0.5022 | 0.0358 | 24.32 | <0.0001 |
|  | Error | 60 | 0.0885 | 0.0014 |  |  |
|  | Total | 74 | 0.5907 |  |  |  |

**Supplementary file 3. ANOVA of female sex pheromones in DGRP lines containing the *Desat2* ancestral allele (ins/ins or ins/del).** df: degrees of freedom; *P*: *P*-value.
