# Supplementary material for: Genetic architecture of natural variation in cuticular hydrocarbon composition in Drosophila melanogaster
Source: eLife. 2015 Nov 14;4:e09861. doi: 10.7554/eLife.09861 (PMC4749392; doi:10.7554/eLife.09861)
Supplement: Supplementary file 5. — DOI: http://dx.doi.org/10.7554/eLife.09861.044 [file elife-09861-supp5.docx]

**Supplementary file 5. Color and symbol codes for DGRP lines used in Figure 5.**

1 = DGRP-21, 2 = DGRP-26,3 = DGRP-28,4 = DGRP-31,5 = DGRP-38,6 = DGRP-40,7 = DGRP-41,8 = DGRP-42,9 = DGRP-45,0 = DGRP-48,A = DGRP-49,B = DGRP-57,C = DGRP-59,D = DGRP-69 (F),E = DGRP-73,F = DGRP-75,G = DGRP-83,H = DGRP-85,I = DGRP-88,J = DGRP-91,K = DGRP-93,L = DGRP-100,M = DGRP-101,N = DGRP-105,O = DGRP-109,P = DGRP-129,Q = DGRP-136,R = DGRP-138,S = DGRP-142,T = DGRP-149,U = DGRP-153,V = DGRP-158,W = DGRP-161,X = DGRP-176,Y = DGRP-177,Z = DGRP-181,a = DGRP-189,b = DGRP-195,c = DGRP-208,d = DGRP-217,e = DGRP-223,f = DGRP-227,g = DGRP-228,h = DGRP-229,i = DGRP-233,j = DGRP-235,k = DGRP-237,l = DGRP-239,m = DGRP-256,n = DGRP-280,o = DGRP-287,p = DGRP-301,q = DGRP-303,r = DGRP-304,s = DGRP-306,t = DGRP-307,u = DGRP-309,v = DGRP-310,w = DGRP-313,x = DGRP-315,y = DGRP-318,z = DGRP-320,+ = DGRP-321,x = DGRP-324,□ = DGRP-332,◊ = DGRP-335,∆ = DGRP-336,Ү = DGRP-338 (F),ᆷ = DGRP-340 (F),ﾛ = DGRP-350 (F),* = DGRP-352 (F),● = DGRP-356 (F),▄ = DGRP-357 (F),▌ = DGRP-358 (F),■ = DGRP-359 (F),♦ = DGRP-360 (F),▽ = DGRP-361 (F),◁ = DGRP-362 (F),▹ = DGRP-365 (F),▲ = DGRP-367,▼ = DGRP-370,◄ = DGRP-371,► = DGRP-373,︿ = DGRP-374,﹀ = DGRP-375,< = DGRP-377,> = DGRP-379,I = DGRP-380,- = DGRP-381,/ = DGRP-382,\ = DGRP-383,1 = DGRP-385,2 = DGRP-386,3 = DGRP-391,4 = DGRP-392,5 = DGRP-399,6 = DGRP-405,7 = DGRP-406,8 = DGRP-409,9 = DGRP-426,0 = DGRP-427,A = DGRP-437,B = DGRP-439,C = DGRP-440,D = DGRP-441,E = DGRP-443,F = DGRP-486,G = DGRP-491,H = DGRP-502,I = DGRP-508,J = DGRP-509,K = DGRP-517,L = DGRP-530,M = DGRP-531,N = DGRP-535,O = DGRP-551,P = DGRP-555,Q = DGRP-559,R = DGRP-563,S = DGRP-584,T = DGRP-589,U = DGRP-595,V = DGRP-639,W = DGRP-642,X = DGRP-646,Y = DGRP-703,Z = DGRP-705,a = DGRP-707,b = DGRP-712,c = DGRP-714,d = DGRP-716,e = DGRP-721,f = DGRP-727,g = DGRP-730,h = DGRP-732,i = DGRP-737,j = DGRP-738,k = DGRP-748,l = DGRP-761,m = DGRP-765,n = DGRP-774,o = DGRP-776,p = DGRP-783,q = DGRP-786,r = DGRP-787,s = DGRP-790,t = DGRP-796,u = DGRP-799,v = DGRP-801,w = DGRP-802,x = DGRP-804,y = DGRP-805,z = DGRP-808,+ = DGRP-810,x = DGRP-812,□ = DGRP-818,◊ = DGRP-819,∆ = DGRP-820,Ү = DGRP-821,ᆷ = DGRP-822,ﾛ = DGRP-832,* = DGRP-837,● = DGRP-843,▄ = DGRP-849,▌ = DGRP-850,■ = DGRP-852,♦ = DGRP-853,▽ = DGRP-855,◁ = DGRP-861,▲ = DGRP-913 (M),,
