# Supplementary material for: Genetic architecture of natural variation in cuticular hydrocarbon composition in Drosophila melanogaster
Source: eLife. 2015 Nov 14;4:e09861. doi: 10.7554/eLife.09861 (PMC4749392; doi:10.7554/eLife.09861)
Supplement: Supplementary file 6. — df: degrees of freedom; SS: Type III sums of squares; F: F statistic; AIC: Akaike information criterion. ***p<0.001; **p<0.01; *p<0.05. DOI: http://dx.doi.org/10.7554/eLife.09861.045 [file elife-09861-supp6.docx]

**Supplementary file 6.** **ANOVA of the effects of *Wolbachia* infection and common polymorphic inversions on CHC PCs**. df: degrees of freedom; SS: Type III sums of squares; F: F statistic; AIC: Akaike information criterion. ***: *P* < 0.001; **: *P* < 0.01; *: *P* < 0.05.

| Analysis | Covariates | df | SS | Residual SS | AIC | F | *P*-value |
| --- | --- | --- | --- | --- | --- | --- | --- |
| F PC1 | none |  |  | 0.72 | -898.46 |  |  |
|  | *Wolbachia* | 1 | 3.20E-05 | 0.72 | -900.45 | 0.01 | 9.34E-01 |
|  | *In(2L)t* | 2 | 4.42E-02 | 0.76 | -892.40 | 4.82 | 9.34E-03** |
|  | *In(2R)NS* | 2 | 1.78E-03 | 0.72 | -902.04 | 0.19 | 8.24E-01 |
|  | *In(3R)P* | 2 | 1.21E-02 | 0.73 | -899.64 | 1.32 | 2.70E-01 |
|  | *In(3R)K* | 2 | 2.41E-01 | 0.96 | -853.74 | 26.23 | 1.49E-10*** |
|  | *In(3R)Mo* | 2 | 3.19E-03 | 0.72 | -901.71 | 0.35 | 7.07E-01 |
| F PC2 | none |  |  | 0.54 | -948.56 |  |  |
|  | *Wolbachia* | 1 | 3.00E-06 | 0.54 | -950.56 | 0.00 | 9.75E-01 |
|  | *In(2L)t* | 2 | 2.23E-02 | 0.56 | -945.68 | 3.26 | 4.08E-02* |
|  | *In(2R)NS* | 2 | 1.03E-02 | 0.55 | -949.34 | 1.51 | 2.23E-01 |
|  | *In(3R)P* | 2 | 2.02E-02 | 0.56 | -946.32 | 2.96 | 5.49E-02 |
|  | *In(3R)K* | 2 | 1.05E-01 | 0.64 | -922.21 | 15.45 | 7.53E-07*** |
|  | *In(3R)Mo* | 2 | 1.97E-02 | 0.56 | -946.45 | 2.89 | 5.85E-02 |
| F PC3 | none |  |  | 0.31 | -1038.50 |  |  |
|  | *Wolbachia* | 1 | 4.60E-05 | 0.31 | -1040.50 | 0.02 | 8.80E-01 |
|  | *In(2L)t* | 2 | 3.50E-03 | 0.32 | -1040.60 | 0.87 | 4.19E-01 |
|  | *In(2R)NS* | 2 | 7.91E-04 | 0.32 | -1042.10 | 0.20 | 8.21E-01 |
|  | *In(3R)P* | 2 | 5.67E-03 | 0.32 | -1039.50 | 1.42 | 2.46E-01 |
|  | *In(3R)K* | 2 | 3.49E-02 | 0.35 | -1024.70 | 8.70 | 2.61E-04*** |
| F PC4 | none |  |  | 0.14 | -1172.50 |  |  |
|  | *Wolbachia* | 1 | 4.05E-04 | 0.14 | -1174.00 | 0.45 | 5.05E-01 |
|  | *In(2L)t* | 2 | 2.41E-03 | 0.14 | -1173.70 | 1.33 | 2.68E-01 |
|  | *In(2R)NS* | 2 | 2.84E-04 | 0.14 | -1176.20 | 0.16 | 8.55E-01 |
|  | *In(3R)P* | 2 | 8.38E-04 | 0.14 | -1175.50 | 0.46 | 6.31E-01 |
|  | *In(3R)K* | 2 | 4.93E-03 | 0.15 | -1170.80 | 2.72 | 6.90E-02 |
|  | *In(3R)Mo* | 2 | 9.80E-04 | 0.14 | -1175.40 | 0.54 | 5.83E-01 |
| F PC5 | none |  |  | 0.07 | -1282.90 |  |  |
|  | *Wolbachia* | 1 | 1.14E-03 | 0.08 | -1282.30 | 2.42 | 1.22E-01 |
|  | *In(2L)t* | 2 | 3.53E-04 | 0.07 | -1286.10 | 0.37 | 6.88E-01 |
|  | *In(2R)NS* | 2 | 6.04E-05 | 0.07 | -1286.70 | 0.06 | 9.38E-01 |
|  | *In(3R)P* | 2 | 3.98E-04 | 0.07 | -1286.00 | 0.42 | 6.56E-01 |
|  | *In(3R)K* | 2 | 5.70E-05 | 0.07 | -1286.80 | 0.06 | 9.41E-01 |
|  | *In(3R)Mo* | 2 | 4.17E-05 | 0.07 | -1286.80 | 0.04 | 9.57E-01 |
| F PC6 | none |  |  | 0.05 | -1333.60 |  |  |
|  | *Wolbachia* | 1 | 4.00E-04 | 0.06 | -1334.40 | 1.15 | 2.86E-01 |
|  | *In(2L)t* | 2 | 5.52E-04 | 0.06 | -1335.90 | 0.79 | 4.56E-01 |
|  | *In(2R)NS* | 2 | 5.42E-04 | 0.06 | -1336.00 | 0.78 | 4.62E-01 |
|  | *In(3R)P* | 2 | 2.27E-04 | 0.06 | -1336.90 | 0.32 | 7.23E-01 |
|  | *In(3R)K* | 2 | 4.16E-04 | 0.06 | -1336.30 | 0.60 | 5.52E-01 |
|  | *In(3R)Mo* | 2 | 1.30E-04 | 0.05 | -1337.20 | 0.19 | 8.30E-01 |
|  |  |  |  |  |  |  |  |
| F PC7 | none |  |  | 0.03 | -1439.20 |  |  |
|  | *Wolbachia* | 1 | 2.69E-04 | 0.03 | -1439.60 | 1.44 | 2.32E-01 |
|  | *In(2L)t* | 2 | 7.99E-05 | 0.03 | -1442.70 | 0.21 | 8.08E-01 |
|  | *In(2R)NS* | 2 | 1.06E-03 | 0.03 | -1437.20 | 2.83 | 6.19E-02 |
|  | *In(3R)P* | 2 | 8.64E-05 | 0.03 | -1442.70 | 0.23 | 7.94E-01 |
|  | *In(3R)K* | 2 | 2.93E-04 | 0.03 | -1441.50 | 0.78 | 4.58E-01 |
|  | *In(3R)Mo* | 2 | 6.70E-04 | 0.03 | -1439.30 | 1.79 | 1.70E-01 |
| M PC1 | none |  |  | 2.02 | -659.46 |  |  |
|  | *Wolbachia* | 1 | 1.76E-02 | 2.04 | -660.09 | 1.27 | 2.62E-01 |
|  | *In(2L)t* | 2 | 4.03E-01 | 2.42 | -634.87 | 14.48 | 1.85E-06*** |
|  | *In(2R)NS* | 2 | 2.92E-02 | 2.05 | -661.20 | 1.05 | 3.53E-01 |
|  | *In(3R)P* | 2 | 9.39E-02 | 2.11 | -656.33 | 3.37 | 3.72E-02* |
|  | *In(3R)K* | 2 | 8.44E-02 | 2.10 | -657.03 | 3.03 | 5.15E-02 |
|  | *In(3R)Mo* | 2 | 2.68E-02 | 2.05 | -661.39 | 0.96 | 3.85E-01 |
| M PC2 | none |  |  | 0.48 | -884.06 |  |  |
|  | *Wolbachia* | 1 | 2.31E-03 | 0.49 | -885.31 | 0.69 | 4.06E-01 |
|  | *In(2L)t* | 2 | 9.27E-03 | 0.49 | -885.08 | 1.39 | 2.52E-01 |
|  | *In(2R)NS* | 2 | 1.34E-03 | 0.48 | -887.63 | 0.20 | 8.18E-01 |
|  | *In(3R)P* | 2 | 5.12E-03 | 0.49 | -886.41 | 0.77 | 4.66E-01 |
|  | *In(3R)K* | 2 | 1.35E-02 | 0.50 | -883.73 | 2.03 | 1.35E-01 |
|  | *In(3R)Mo* | 2 | 2.74E-03 | 0.49 | -887.18 | 0.41 | 6.64E-01 |
| M PC3 | none |  |  | 0.14 | -1081.50 |  |  |
|  | *Wolbachia* | 1 | 2.94E-04 | 0.14 | -1083.20 | 0.31 | 5.78E-01 |
|  | *In(2L)t* | 2 | 1.08E-03 | 0.14 | -1084.30 | 0.57 | 5.68E-01 |
|  | *In(2R)NS* | 2 | 1.41E-03 | 0.14 | -1083.90 | 0.74 | 4.77E-01 |
|  | *In(3R)P* | 2 | 1.57E-02 | 0.15 | -1068.60 | 8.27 | 3.97E-04*** |
|  | *In(3R)K* | 2 | 2.77E-03 | 0.14 | -1082.40 | 1.46 | 2.36E-01 |
|  | *In(3R)Mo* | 2 | 8.49E-04 | 0.14 | -1084.60 | 0.45 | 6.40E-01 |
| M PC4 | none |  |  | 0.07 | -1191.50 |  |  |
|  | *Wolbachia* | 1 | 1.70E-06 | 0.07 | -1193.50 | 0.00 | 9.52E-01 |
|  | *In(2L)t* | 2 | 2.59E-03 | 0.07 | -1189.70 | 2.76 | 6.69E-02. |
|  | *In(2R)NS* | 2 | 2.83E-04 | 0.07 | -1194.80 | 0.30 | 7.41E-01 |
|  | *In(3R)P* | 2 | 6.37E-05 | 0.07 | -1195.40 | 0.07 | 9.35E-01 |
|  | *In(3R)K* | 2 | 2.53E-04 | 0.07 | -1194.90 | 0.27 | 7.64E-01 |
|  | *In(3R)Mo* | 2 | 3.34E-04 | 0.07 | -1194.70 | 0.36 | 7.01E-01 |
| M PC5 | none |  |  | 0.04 | -1268.20 |  |  |
|  | *Wolbachia* | 1 | 1.70E-04 | 0.04 | -1269.60 | 0.59 | 4.43E-01 |
|  | *In(2L)t* | 2 | 5.67E-05 | 0.04 | -1272.00 | 0.10 | 9.06E-01 |
|  | *In(2R)NS* | 2 | 6.62E-05 | 0.04 | -1272.00 | 0.11 | 8.92E-01 |
|  | *In(3R)P* | 2 | 6.21E-04 | 0.04 | -1269.90 | 1.08 | 3.43E-01 |
|  | *In(3R)K* | 2 | 5.09E-03 | 0.05 | -1254.20 | 8.82 | 2.43E-04*** |
|  | *In(3R)Mo* | 2 | 8.77E-04 | 0.04 | -1269.00 | 1.52 | 2.22E-01 |
