# Supplementary material for: Genetic architecture of natural variation in cuticular hydrocarbon composition in Drosophila melanogaster
Source: eLife. 2015 Nov 14;4:e09861. doi: 10.7554/eLife.09861 (PMC4749392; doi:10.7554/eLife.09861)
Supplement: Supplementary file 10. — DOI: http://dx.doi.org/10.7554/eLife.09861.049 [file elife-09861-supp10.docx]

**Supplementary File 10. Chemical identification of previously unpublished CHC in *Drosophila melanogaster***

Chemical identification of methyl-branched hydrocarbons where the methyl group is near to the chain terminus was based on the relatively high abundance of indicating fragments near the molecular ion. These are produced in the EI ion source by *α*-cleavage at the methyl branch on the side of the near chain terminus. Hydrogen rearrangement also leads to low-weight radical ion fragments with an even weight corresponding to fragmentation on the other side of the methyl branch. Mass spectra and characteristic ions are shown on the figures below, see A and B for 3-methyl- and 5-methylpentacosane, from a male and a female in this study, respectively. Methyl branch position on carbon 3 was also confirmed by the retention index of these hydrocarbons: they all have a retention index increment of 68–73 on the 5% phenyl-methylpolysiloxane column (peaks 17, 41, and 53 in Table 1 in the manuscript) due to the methyl branch at that particular position (Schulz, Lipids 2001, 36:637-647).

Though the position of double bond in monoalkenes was not confirmed with microderivatization reactions the retention indices were matched to that of already published monoalkenes in *D. melanogaster.* Even positions were tentatively assigned when there was a gas-chromatographic peak with a molecular ion and fragmentation pattern of a monoalkene between two peaks of monoalkenes with double bonds at consecutive odd positions, i.e. 6- was assigned in between 7- and 5- (peak 30 between 29 and 31 in Table 1).

As for alkadienes with the double bonds in even positions just a few carbons apart, we followed the same principle as described in Howard et al. (Journal of Chemical Ecology 2003, 29:961-976), namely that these compounds (as the ones with the double bonds in odd positions) have characteristic fragments resulting from the fragmentation of the molecular ion between carbons where the double bonds are and from *α*-cleavage at the outer side of the double bonds. An example on figure C demonstrates the above with 6,10-hexacosadiene from one of the females analyzed in this study.
